# Supplementary material for: Dissipation, Residue and Dietary Intake Risk Assessment of Penthiopyrad in Eggplants and Its Removal Using Various Household Processing Techniques
Source: Foods. 2022 Oct 23;11(21):3327. doi: 10.3390/foods11213327 (PMC9655489; doi:10.3390/foods11213327)
Supplement: Supplementary file 1 [file foods-11-03327-s001.zip › foods-1895136-supplementary.pdf]

Supplementary information

Extraction and purification

10.00 ± 0.05 g of eggplant (raw, steamed and boiled) sample was weighed in a 50 mL centrifuge tube. After adding 20 mL acetonitrile, 2 g NaCl and 4 g anhydrous $NaSO_4$, the mixture was vortexed at a relative centrifugal force (RCF) of 1677 × *g* for 5 min and then centrifuged at a RCF of 4025 × *g* for 3 min. Thereafter, 1 mL of supernatant was transferred to a 2-mL centrifuge tube containing 50 mg of PSA. The mixture was vortex-mixed for 30 s, centrifuged at a RCF of 6708 × *g* for 3 min and filtered through a 0.22 μm syringe nylon filter before liquid chromatography with tandem mass spectrometry (LC-MS/MS) analysis.

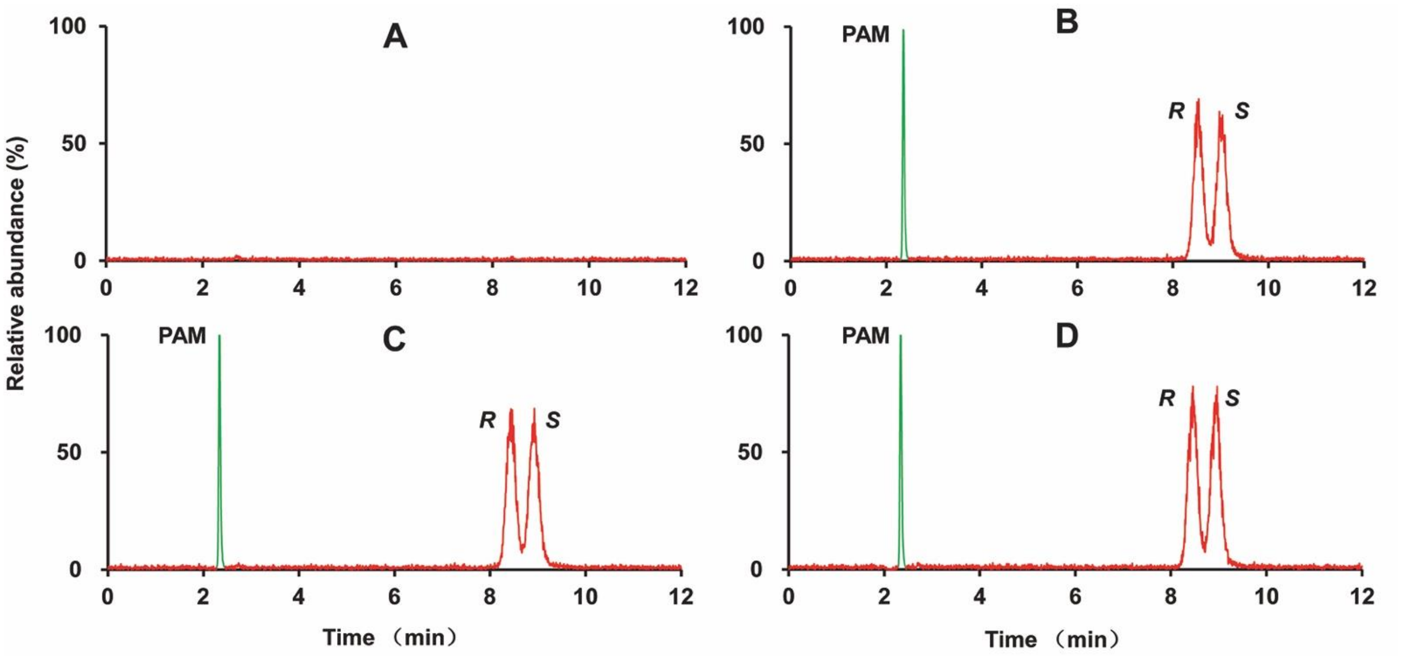

Figure S1. Typical LC-MS/MS chromatograms of penthiopyrad stereoisomers and its metabolite PAM in blank eggplant (A) samples, standard solution (B) (100 μg/kg), spiked (100 μg/kg) eggplant (C) samples, actual eggplant (D) samples.

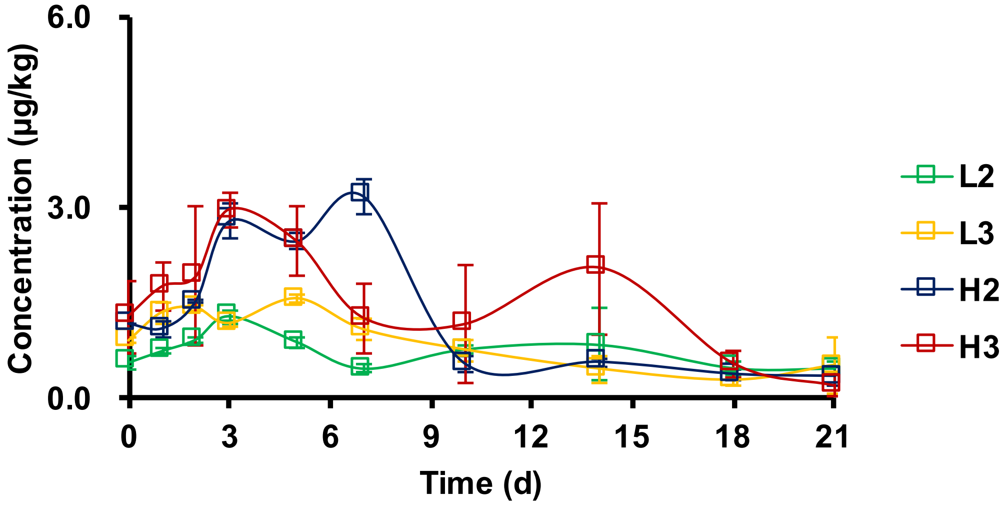

Figure S2. Residue levels of penthiopyrad metabolite PAM in eggplant samples under field conditions. Error bars represent standard deviation (SD) of triplicate measurements. L2, L3, H2 and H3 represent spraying dose of 75 g a.i./ha twice and three times, and dose of 99 g a.i./ha twice and three times, respectively.

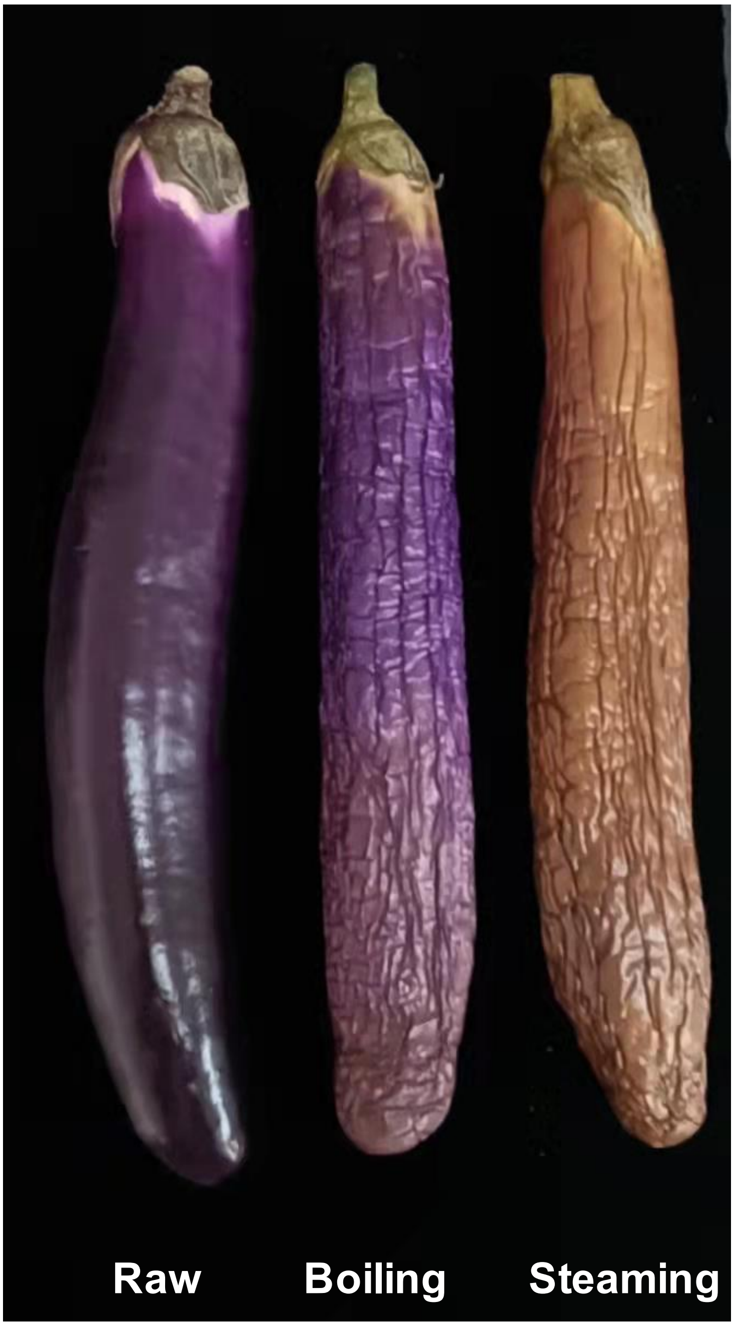

Figure S3. Appearance of eggplant after different treatments.

Table S1. LC-MS/MS parameters for detecting penthiopyrad stereoisomers and its metabolite PAM.

Parameter	Data
LC conditions (UFLC DGU-20A LC system, Shimadzu Corporation, Kyoto, Japan)	
Column	Superchiral S-OD chiral column (150 × 4.6 mm i.d.; particle size, 5 μm; Shanghai Chiralway Biotech Co., Ltd., Shanghai, China)
Mobile phase	Acetonitrile (0.1% formic acid):0.1% formic acid aqueous solution = 50:50 (*v*/*v*)
Flow rate	1.0 mL/min
Injection volume	3 μL
Column temperature	30 °C
Running time	11 min
MS conditions (Sciex 4000 Q-Trap triple quadrupole MS system, Applied Biosystems, Foster City, USA)	
Gas source	Nitrogen (purity, 99.999%)
Pressure of ion source gas 1 and 2	65 psi
Ion spray voltage	5500 V
Ion source temperature	600 °C
Ionization mode	Positive electron spray ionization (ESI^+)
Monitoring mode	Multiple reaction monitoring (MRM)
m/*z*	Penthiopyrad: 360.3→256.0 (confirmation), 360.3→276.0 (quantification)<br>PAM: 194.2→134.0 (confirmation), 194.2→174.2 (quantification)
Declustering potential (DP)	Penthiopyrad: 103.5 V<br>PAM: 57.1 V
Entrance potential (EP)	10.0 V
Collision energy (CE)	Penthiopyrad: 28.7 (daughter ion 256.0), 18.4 (daughter ion 276.0)<br>PAM: 26.8 (daughter ion 134.0), 14.2 (daughter ion 174.2)
Collision cell exit potential (CXP)	Penthiopyrad: 15.7 (daughter ion 256.0), 25.9 (daughter ion 276.0)<br>PAM: 11.0 (daughter ion 134.0), 11.0 (daughter ion 174.2)

Table S2. Quality control of actual field eggplant sample detection.

Matrix	Analyte	Spiked level (μg/kg)	Average recovery, RSD (%, n = 3)
Eggplant/raw	*R*-penthiopyrad	100	91.1, 3.4
	S-penthiopyrad		91.2, 1.7
	PAM		84.6, 4.6
Eggplant/steamed	*R*-penthiopyrad		92.0, 2.1
	S-penthiopyrad		91.5, 3.1
	PAM		84.6, 4.1
Eggplant/boiled	*R*-penthiopyrad		91.8, 4.2
	S-penthiopyrad		92.4, 2.6
	PAM		85.0, 1.5

Table S3. Residue levels and dissipation percentages of *R*-penthiopyrad in eggplant samples under field conditions.

**Residue levels (μg/kg)**									
**Dose (g a.i./ha)**		**75**				**99**			
**Spraying time**		**2**	P_1	**3**	P_2	**2**	P_3	**3**	P_4
Interval(d)	0	59.0±3.1a	< 0.001	78.7±2.7a	< 0.001	172.1±4.9a	< 0.001	169.6±3.9a	< 0.001
	1	41.3±3.3b		65.4±4.6b		159.9±10.7a		108.4±5.9b	
	2	28.8±5.4c		60.9±9.9b		76.3±16.8b		96.5±2.7c	
	3	18.2±0.7d		16.5±1.1c		38.0±2.9c		55.3±8.1d	
	5	7.1±1.8e		15.3±1.4c		28.8±4.0cd		48.0±2.8d	
	7	3.0±0.5ef		4.7±0.5d		16.1±1.9de		28.3±7.3e	
	10	1.1±0.2f		4.0±0.2d		2.4±0.2e		7.4±1.1f	
	14	0.7±0f		0.2±0d		0.9±0.1e		2.4±0.2f	
	18	0.3±0f		0.1±0d		0.2±0.1e		0.8±0.2f	
	21	0.2±0.1f		0.1±0d		0.1±0e		0.7±0f	
**Dissipation percentage (%)**									
**Dose (g a.i./ha)**		**75**				**99**			
**Spraying time**		**2**	P_1	**3**	P_2	**2**	P_3	**3**	P_4
Interval(d)	0	/	< 0.001	/	< 0.001	/	< 0.001	/	< 0.001
	1	30.2±3.2e		17.0±5.5c		7.2±4.6e		36.0±4.9e	
	2	51.5±8.6d		22.9±13.6c		55.9±10.6d		43.0±3.5d	
	3	69.2±1.2c		79.0±1.1b		77.9±1.3c		67.5±5.0c	
	5	88.1±3.1b		80.6±1.4b		83.3±2.3bc		71.7±1.5c	
	7	94.9±0.7a		94.1±0.5a		90.7±1.0b		83.4±5.1b	
	10	98.1±0.4a		94.8±0.2a		98.6±0.1a		95.7±0.7a	
	14	98.9±0a		99.8±0a		99.5±0.1a		98.6±0.1a	
	18	99.5±0a		99.8±0.1a		99.9±0.1a		99.5±0.1a	
	21	99.7±0.1a		99.9±0.1a		99.9±0a		99.6±0a	

Data are expressed as average values ± SD. P_1, P_2, P_3 and P_4 represent confidence within the 99% confidence level of EF values of spraying dose of 75 g a.i./ha twice and three times, dose of 99 g a.i./ha twice and three times, respectively; different lower case letters indicate statistical significance between different intervals for each dose according to Duncan's multiple range test ($P = 0.01$).

Table S4. Residue levels and dissipation percentages of *S*-penthiopyrad in eggplant samples under field conditions.

**Residue levels (μg/kg)**									
**Dose (g a.i./ha)**		**75**				**99**			
**Spraying time**		**2**	P_1	**3**	P_2	**2**	P_3	**3**	P_4
Interval(d)	0	58.5±3.3a	< 0.001	78.8±2.3a	< 0.001	166.8±1.9a	< 0.001	170.0±4.0a	< 0.001
	1	40.9±2.4b		65.3±4.5b		157.4±11.8a		106.8±5.4b	
	2	28.5±5.2c		60.2±9.3b		83.0±7.8b		94.9±2.1c	
	3	18.1±0.9d		17.0±0.7c		39.0±3.2c		55.0±8.4d	
	5	7.3±1.8e		15.7±1.6c		28.5±4.3cd		49.6±3.0d	
	7	3.2±0.5ef		4.9±0.5d		16.7±1.7d		28.4±7.3e	
	10	1.1±0.1f		4.3±0.1d		2.6±0.3e		7.4±1.2f	
	14	0.7±0.1f		0.2±0d		1.0±0.1e		2.4±0.1f	
	18	0.3±0.1f		0.1±0d		0.2±0.1e		0.9±0.2f	
	21	0.2±0.1f		0.1±0d		0.1±0.1e		0.7±0f	
**Dissipation percentage (%)**									
**Dose (g a.i./ha)**		**75**				**99**			
**Spraying time**		**2**	P_1	**3**	P_2	**2**	P_3	**3**	P_4
Interval(d)	0	/	< 0.001	/	< 0.001	/	< 0.001	/	< 0.001
	1	30.0±3.3e		17.2±6.0c		5.7±7.4f		37.2±2.8e	
	2	51.6±8.4d		23.8±12.9c		50.3±5.0e		44.2±1.2d	
	3	69.0±0.7c		78.5±0.6b		76.6±2.0d		67.7±5.9c	
	5	87.6±3.1b		80.2±1.8b		82.9±2.9c		70.8±2.0c	
	7	94.6±0.8a		93.8±0.6a		90.0±1.1b		83.3±4.9b	
	10	98.1±0.1a		94.5±0.1a		98.4±0.2a		95.6±0.8a	
	14	98.8±0.1a		99.8±0a		99.4±0a		98.6±0.1a	
	18	99.4±0.2a		99.8±0a		99.8±0.1a		99.5±0.1a	
	21	99.6±0.1a		99.9±0.1a		99.9±0a		99.6±0a	

Data are expressed as average values ± SD. P_1, P_2, P_3 and P_4 represent confidence within the 99% confidence level of EF values of spraying dose of 75 g a.i./ha twice and three times, dose of 99 g a.i./ha twice and three times, respectively; different lower case letters indicate statistical significance between different intervals for each dose according to Duncan's multiple range test (P = 0.01).

Table S5. EF values of penthiopyrad in eggplant under field conditions.

**EF**									
**Dose (g a.i./ha)**		**75**				**99**			
**Spraying time**		**2**	$\boldsymbol{P_1}$	**3**	$\boldsymbol{P_2}$	**2**	$\boldsymbol{P_3}$	**3**	$\boldsymbol{P_4}$
Interval(d)	0	0.50±0.00a	0.406	0.50±0.00a	0.561	0.51±0.01a	0.030	0.50±0.01a	0.258
	1	0.50±0.01a		0.50±0.00a		0.50±0.00a		0.50±0.03a	
	2	0.50±0.00a		0.50±0.00a		0.47±0.05ab		0.50±0.01a	
	3	0.50±0.01a		0.49±0.01a		0.49±0.00a		0.50±0.00a	
	5	0.49±0.00a		0.49±0.00a		0.50±0.00a		0.49±0.00a	
	7	0.49±0.00a		0.49±0.00a		0.49±0.00ab		0.50±0.00a	
	10	0.49±0.03a		0.48±0.01a		0.48±0.01ab		0.50±0.00a	
	14	0.49±0.02a		0.48±0.01a		0.47±0.02ab		0.49±0.01a	
	18	0.48±0.09a		0.49±0.10a		0.48±0.01ab		0.49±0.01a	
	21	0.43±0.02a		0.43±0.08a		0.45±0.03b		0.48±0.01a	

Data are expressed as average values ± SD. P_1, P_2, P_3 and P_4 represent confidence within the 99% confidence level of EF values of spraying dose of 75 g a.i./ha twice and three times, dose of 99 g a.i./ha twice and three times, respectively; different lower case letters indicate statistical significance between different intervals for each dose according to Duncan's multiple range test (P = 0.01).

Table S6. Final residues of stereoisomeric, racemic and total penthiopyrad in eggplant under field conditions.

Dose (g a.i./ha)	Spraying time	Interval (d)	Final residue (μg/kg)							
			R	P_1	*S*	P_2	*Rac*	P_3	Total	P_4
75	2	5	< 10	< 0.001	< 10	< 0.001	14±4d	< 0.001	16±4d	< 0.001
		7	< 10		< 10		< 10		< 10	
		10	< 10		< 10		< 10		< 10	
		14	< 10		< 10		< 10		< 10	
	3	5	15±1c		16±2c		31±3c		34±3c	
		7	< 10		< 10		10±1d		12±1d	
		10	< 10		< 10		< 10		10±0d	
		14	< 10		< 10		< 10		< 10	
99	2	5	29±4b		28±4b		57±8b		62±8b	
		7	16±2c		17±2c		33±4c		39±3c	
		10	< 10		< 10		< 10		< 10	
		14	< 10		< 10		< 10		< 10	
	3	5	48±3a		50±3a		98±6a		102±5a	
		7	28±7b		28±7b		57±15b		59±14b	
		10	< 10		< 10		15±2d		17±4d	
		14	< 10		< 10		< 10		< 10	

Data are expressed as average values ± SD. P_1, P_2, P_3 and P_4 represent confidence within the 99% confidence level of *R*-(−)-stereoisomer, *S*-(+)-stereoisomer, *rac*-penthiopyrad and total penthiopyrad (sum of *rac*-penthiopyrad and PAM), respectively; different lower case letters indicate statistical significance between different treatments for eggplant under field conditions according to Duncan's multiple range test (P = 0.01).

Table S7. Acute risk assessment of stereoisomeric, racemic and total penthiopyrad in eggplant under field conditions.

Age	Sex	b.w. (kg)	Dark vegetable (g/d)	RQa (%, Average ± SD, *n* = 3)															
				14 d				10 d				7 d				5 d			
				R	*S*	*Rac*	Total	*R*	*S*	*Rac*	Total	*R*	*S*	*Rac*	Total	*R*	*S*	*Rac*	Total
2-3	M	13.2	43	0±0	0±0	0±0	0±0	0±0	0±0	0±0	0±0	0±0	0±0	0±0	0±0	0±0	0±0	0±0	0±0
	F	12.3	39.6	0±0	0±0	0±0	0±0	0±0	0±0	0±0	0±0	0±0	0±0	0±0	0±0	0±0	0±0	0±0	0±0
4-6	M	16.8	56.4	0±0	0±0	0±0	0±0	0±0	0±0	0±0	0±0	0±0	0±0	0±0	0±0	0±0	0±0	0±0	0±0
	F	16.2	56.2	0±0	0±0	0±0	0±0	0±0	0±0	0±0	0±0	0±0	0±0	0±0	0±0	0±0	0±0	0±0	0±0
7-10	M	22.9	70.2	0±0	0±0	0±0	0±0	0±0	0±0	0±0	0±0	0±0	0±0	0±0	0±0	0±0	0±0	0±0	0±0
	F	21.7	65.9	0±0	0±0	0±0	0±0	0±0	0±0	0±0	0±0	0±0	0±0	0±0	0±0	0±0	0±0	0±0	0±0
11-13	M	34.1	77.2	0±0	0±0	0±0	0±0	0±0	0±0	0±0	0±0	0±0	0±0	0±0	0±0	0±0	0±0	0±0	0±0
	F	34	73.1	0±0	0±0	0±0	0±0	0±0	0±0	0±0	0±0	0±0	0±0	0±0	0±0	0±0	0±0	0±0	0±0
14-17	M	46.7	87.1	0±0	0±0	0±0	0±0	0±0	0±0	0±0	0±0	0±0	0±0	0±0	0±0	0±0	0±0	0±0	0±0
	F	45.2	81.5	0±0	0±0	0±0	0±0	0±0	0±0	0±0	0±0	0±0	0±0	0±0	0±0	0±0	0±0	0±0	0±0
18-29	M	58.4	92.1	0±0	0±0	0±0	0±0	0±0	0±0	0±0	0±0	0±0	0±0	0±0	0±0	0±0	0±0	0±0	0±0
	F	52.1	84.5	0±0	0±0	0±0	0±0	0±0	0±0	0±0	0±0	0±0	0±0	0±0	0±0	0±0	0±0	0±0	0±0
30-44	M	64.9	93.7	0±0	0±0	0±0	0±0	0±0	0±0	0±0	0±0	0±0	0±0	0±0	0±0	0±0	0±0	0±0	0±0
	F	55.7	91.3	0±0	0±0	0±0	0±0	0±0	0±0	0±0	0±0	0±0	0±0	0±0	0±0	0±0	0±0	0±0	0±0
45-59	M	63.1	99.5	0±0	0±0	0±0	0±0	0±0	0±0	0±0	0±0	0±0	0±0	0±0	0±0	0±0	0±0	0±0	0±0
	F	57	94.7	0±0	0±0	0±0	0±0	0±0	0±0	0±0	0±0	0±0	0±0	0±0	0±0	0±0	0±0	0±0	0±0
60-69	M	61.5	97.7	0±0	0±0	0±0	0±0	0±0	0±0	0±0	0±0	0±0	0±0	0±0	0±0	0±0	0±0	0±0	0±0
	F	54.3	93.2	0±0	0±0	0±0	0±0	0±0	0±0	0±0	0±0	0±0	0±0	0±0	0±0	0±0	0±0	0±0	0±0
≥70	M	58.5	88.6	0±0	0±0	0±0	0±0	0±0	0±0	0±0	0±0	0±0	0±0	0±0	0±0	0±0	0±0	0±0	0±0
	F	51	75.3	0±0	0±0	0±0	0±0	0±0	0±0	0±0	0±0	0±0	0±0	0±0	0±0	0±0	0±0	0±0	0±0

M, male; F, female; b.w., body weight; RQa, acute risk quotient; *R*, *R*-(–)-stereoisomer; *S*, *S*-(+)-stereoisomer; *Rac*, *rac*-penthiopyrad; Total, sum of *rac*-penthiopyrad and PAM.

Table S8. Chronic risk assessment of stereoisomeric, racemic and total penthiopyrad in eggplant under field conditions.

**Age**	**Sex**	**b.w. (kg)**	**Dark vegetable (g/d)**	**RQ_c (%, Average ± SD, *n* = 3)**															
				**14 d**				**10 d**				**7 d**				**5 d**			
				R	***S***	***Rac***	**Total**	***R***	***S***	***Rac***	**Total**	***R***	***S***	***Rac***	**Total**	***R***	***S***	*Rac*	Total
2-3	M	13.2	43	0±0	0±0	0±0	0±0	0±0	0±0	0±0	0±0	0±0	0±0	0.1±0.1	0.1±0.1	0.1±0.1	0.1±0.1	0.2±0.1	0.2±0.1
	F	12.3	39.6	0±0	0±0	0±0	0±0	0±0	0±0	0±0	0±0	0±0	0±0	0.1±0.1	0.1±0.1	0.1±0.1	0.1±0.1	0.2±0.1	0.2±0.1
4-6	M	16.8	56.4	0±0	0±0	0±0	0±0	0±0	0±0	0±0	0±0	0±0	0±0	0.1±0.1	0.1±0.1	0.1±0.1	0.1±0.1	0.2±0.1	0.2±0.1
	F	16.2	56.2	0±0	0±0	0±0	0±0	0±0	0±0	0±0	0±0	0±0	0±0	0.1±0.1	0.1±0.1	0.1±0.1	0.1±0.1	0.2±0.1	0.2±0.1
7-10	M	22.9	70.2	0±0	0±0	0±0	0±0	0±0	0±0	0±0	0±0	0±0	0±0	0.1±0.1	0.1±0.1	0.1±0.1	0.1±0.1	0.1±0.1	0.2±0.1
	F	21.7	65.9	0±0	0±0	0±0	0±0	0±0	0±0	0±0	0±0	0±0	0±0	0.1±0.1	0.1±0.1	0.1±0.1	0.1±0.1	0.1±0.1	0.2±0.1
11-13	M	34.1	77.2	0±0	0±0	0±0	0±0	0±0	0±0	0±0	0±0	0±0	0±0	0.1±0.1	0.1±0.1	0.1±0	0.1±0	0.1±0.1	0.1±0.1
	F	34	73.1	0±0	0±0	0±0	0±0	0±0	0±0	0±0	0±0	0±0	0±0	0.1±0.1	0.1±0.1	0.1±0	0.1±0	0.1±0.1	0.1±0.1
14-17	M	46.7	87.1	0±0	0±0	0±0	0±0	0±0	0±0	0±0	0±0	0±0	0±0	0.1±0	0.1±0.1	0±0	0±0	0.1±0.1	0.1±0.1
	F	45.2	81.5	0±0	0±0	0±0	0±0	0±0	0±0	0±0	0±0	0±0	0±0	0±0	0.1±0.1	0±0	0±0	0.1±0.1	0.1±0.1
18-29	M	58.4	92.1	0±0	0±0	0±0	0±0	0±0	0±0	0±0	0±0	0±0	0±0	0±0	0±0	0±0	0±0	0.1±0.1	0.1±0.1
	F	52.1	84.5	0±0	0±0	0±0	0±0	0±0	0±0	0±0	0±0	0±0	0±0	0±0	0.1±0.0	0±0	0±0	0.1±0.1	0.1±0.1
30-44	M	64.9	93.7	0±0	0±0	0±0	0±0	0±0	0±0	0±0	0±0	0±0	0±0	0±0	0±0	0±0	0±0	0.1±0.1	0.1±0.1
	F	55.7	91.3	0±0	0±0	0±0	0±0	0±0	0±0	0±0	0±0	0±0	0±0	0±0	0.1±0.0	0±0	0±0	0.1±0.1	0.1±0.1
45-59	M	63.1	99.5	0±0	0±0	0±0	0±0	0±0	0±0	0±0	0±0	0±0	0±0	0±0	0±0	0±0	0±0	0.1±0.1	0.1±0.1
	F	57	94.7	0±0	0±0	0±0	0±0	0±0	0±0	0±0	0±0	0±0	0±0	0±0	0.1±0.0	0±0	0±0	0.1±0.1	0.1±0.1
60-69	M	61.5	97.7	0±0	0±0	0±0	0±0	0±0	0±0	0±0	0±0	0±0	0±0	0±0	0.1±0.0	0±0	0±0	0.1±0.1	0.1±0.1
	F	54.3	93.2	0±0	0±0	0±0	0±0	0±0	0±0	0±0	0±0	0±0	0±0	0±0	0.1±0.0	0±0	0±0	0.1±0.1	0.1±0.1
≥70	M	58.5	88.6	0±0	0±0	0±0	0±0	0±0	0±0	0±0	0±0	0±0	0±0	0±0	0±0	0±0	0±0	0.1±0.1	0.1±0.1
	F	51	75.3	0±0	0±0	0±0	0±0	0±0	0±0	0±0	0±0	0±0	0±0	0±0	0±0	0±0	0±0	0.1±0.1	0.1±0.1

M, male; F, female; b.w., body weight; RQ_c, chronic risk quotient; *R*, *R*-(–)-stereoisomer; *S*, *S*-(+)-stereoisomer; *Rac*, *rac*-penthiopyrad; Total, sum of *rac*-penthiopyrad and PAM.

Table S9. Reduction ratios (RRs) of penthiopyrad from eggplant under different household processing conditions.

Processing variety		Number	Concentration	Time/min	RR (*R*)	P_1	RR (*S*)	P_2	RR (*Rac*)	P_3
Washing	Tap water rinsing	W1		30	93.5±0.5%a	< 0.001	93.6±0.5%a	< 0.001	93.5±0.5%a	< 0.001
	Stir and soaking	W2		30	74.9±0.4%b		75.4±0.7%b		75.1±0.5%b	
	Static and soaking	W3		30	62.5±5.7%c		65.0±4.8%c		63.8±5.3%c	
	Sodium bicarbonate	W4	1%	30	99.3±0.1%a	< 0.001	99.3±0.2%a	< 0.001	99.3±0.1%a	< 0.001
			0.5%		97.9±0.6%b		98.0±0.5%b		98.0±0.6%b	
			0.2%		84.6±3.8%c		85.1±3.8%c		84.9±3.8%c	
	Acetic acid	W5	1%	30	97.3±0.3%a	< 0.001	97.3±0.3%a	< 0.001	97.3±0.3%a	< 0.001
			0.5%		91.1±1.7%b		90.8±2.9%b		90.9±2.2%b	
			0.2%		55.3±4.3%c		58.3±4.0%c		56.8±4.2%c	
	Sodium chloride	W6	1%	30	97.8±1.0%a	< 0.001	97.8±0.9%a	< 0.001	97.8±0.9%a	< 0.001
			0.5%		93.2±0.5%b		93.0±0.6%b		93.1±0.5%b	
			0.2%		89.3±1.9%c		90.0±0.8%c		89.7±1.3%c	
	Ethanol	W7	1%	30	99.1±0.1%a	< 0.001	99.2±0.1%a	< 0.001	99.2±0.1%a	< 0.001
			0.5%		96.1±1.1%b		96.2±1.0%b		96.1±1.1%b	
			0.2%		87.3±0.5%c		87.8±0.4%c		87.5±0.5%c	
	Sodium dodecyl benzene sulfonate	W8	1%	30	99.6±0.1%a	< 0.001	99.6±0.1%a	< 0.001	99.6±0.1%a	< 0.001
			0.5%		94.1±1.1%b		94.3±1.0%b		94.2±1.1%b	
			0.2%		91.2±0.9%c		91.5±0.9%c		91.4±0.9%c	
Peeling	Raw	P1			99.6±0%c	< 0.001	99.6±0%c	< 0.001	99.6±0%c	< 0.001
	Steaming	P2		10	99.8±0.1%a		99.8±0.1%a		99.8±0.1%a	
	Boiling	P3		10	99.7±0.1%b		99.7±0.1%b		99.7±0.1%b	
Steaming		S1		5	96.1±0.4%c	< 0.001	96.1±0.5%c	< 0.001	96.1±0.4%c	< 0.001
		S2		10	98.8±0.1%b		98.8±0.1%b		98.8±0.1%b	
		S3		20	99.1±0.1%a		99.0±0.1%a		99.0±0.1%a	
Boiling		B1		5	40.3±2.1%c	< 0.001	42.1±2.3%c	< 0.001	41.2±2.2%c	< 0.001
		B2		10	48.5±3.5%b		50.1±3.2%b		49.4±3.3%b	
		B3		20	69.9±9.6%a		70.5±9.4%a		70.2±9.5%a	

Data are expressed as average values ± SD. P_1, P_2 and P_3 represent confidence within the 99% confidence level of *R*-(−)-stereoisomer, *S*-(+)-stereoisomer, *rac*-penthiopyrad, respectively; different lower case letters indicate statistical significance between different treatments for processed eggplant samples according to Duncan's multiple range test ($P = 0.01$).
